# The ATP-P2X_7_ Signaling Axis Is an Essential Sentinel for Intracellular *Clostridium difficile* Pathogen-Induced Inflammasome Activation

**DOI:** 10.3389/fcimb.2018.00084

**Published:** 2018-03-16

**Authors:** Ya-Hui Liu, Yung-Chi Chang, Liang-Kuei Chen, Po-An Su, Wen-Chien Ko, Yau-Sheng Tsai, Yi-Hsuan Chen, Hsin-Chih Lai, Cheng-Yeu Wu, Yuan-Pin Hung, Pei-Jane Tsai

**Affiliations:** ^1^Department of Medical Laboratory Science and Biotechnology, College of Medicine, National Cheng Kung University, Tainan, Taiwan; ^2^Department of Pathology, National Cheng Kung University Hospital, Tainan, Taiwan; ^3^Division of Infectious Diseases, Chi Mei Medical Center, Tainan, Taiwan; ^4^Department of Pharmacy, Chia Nan University of Pharmacy and Science, Tainan, Taiwan; ^5^Department of Internal Medicine, National Cheng Kung University Hospital, Tainan, Taiwan; ^6^Center for Infection Control, National Cheng Kung University Hospital, Tainan, Taiwan; ^7^Department of Medicine, College of Medicine, National Cheng Kung University, Tainan, Taiwan; ^8^Institute of Clinical Medicine, College of Medicine, National Cheng Kung University, Tainan, Taiwan; ^9^Cardiovascular Research Center, College of Medicine, National Cheng Kung University, Tainan, Taiwan; ^10^Department of Medical Laboratory Science and Biotechnology, Chang Gung University, Taoyaun, Taiwan; ^11^Research Center for Industry of Human Ecology, College of Human Ecology, Chang Gung University of Science and Technology, Taoyaun, Taiwan; ^12^Graduate Institute of Health Industry and Technology, College of Human Ecology, Chang Gung University of Science and Technology, Taoyaun, Taiwan; ^13^Center for Molecular and Clinical Immunology, Chang Gung University, Taoyaun, Taiwan; ^14^Research Center of Bacterial Pathogenesis, Chang Gung University, Taoyaun, Taiwan; ^15^Department of Internal Medicine, Tainan Hospital, Ministry of Health and Welfare, Tainan, Taiwan; ^16^Center of Infectious Disease and Signaling Research, National Cheng Kung University, Tainan, Taiwan

**Keywords:** *Clostridium difficile*, inflammasome activation, pyroptosis, ATP-P2X_7_ pathway, MyD88

## Abstract

*Clostridium difficile* infection (CDI) is the leading cause of nosocomial infection in hospitalized patients receiving long-term antibiotic treatment. An excessive host inflammatory response is believed to be the major mechanism underlying the pathogenesis of *C. difficile* infection, and various proinflammatory cytokines such as IL-1β are detected in patients with *C. difficile* infection. IL-1β is known to be processed by caspase-1, a cysteine protease that is regulated by a protein complex called the inflammasome, which leads to a specialized form of cell death called pyroptosis. The function of inflammasome activation-induced pyroptosis is to clear or limit the spread of invading pathogens via infiltrated neutrophils. Here, we focused on inflammasome activation induced by intact *C. difficile* to re-evaluate the nature of inflammasome activation in CDI pathogenesis, which could provide information that leads to an alternative therapeutic strategy for the treatment of this condition in humans. First, we found that caspase-1-dependent IL-1β production was induced by *C. difficile* pathogens in macrophages and increased in a time-dependent manner. Moreover, intracellular toxigenic *C. difficile* was essential for ATP-P2X_7_ pathway of inflammasome activation and subsequent caspase-1-dependent pyroptotic cell death, leading to the loss of membrane integrity and release of intracellular contents such as LDH. Notably, we also observed that bacterial components such as surface layer proteins (SLPs) were released from pyroptotic cells. In addition, pro-IL-1β production was completely MyD88 and partially TLR2 dependent. Finally, to investigate the role of the caspase-1-dependent inflammasome in host defense, we found that colonic inflammasome activation was also induced by CDI and that caspase-1 inhibition by Ac-YVAD-CMK led to increased disease progression and *C. difficile* load. Taken together, the present results suggest that MyD88 and TLR2 are critical component in pro-IL-1β production and intracellular *C. difficile* following the ATP-P2X_7_ pathway of inflammasome activation and pyroptosis, which play important roles in host defense through the utilization of inflammation-mediated bacterial clearance mechanisms during *C. difficile* infection.

## Introduction

*Clostridium difficile* infection (CDI) is the most well-known condition among the etiologies of nosocomial infectious diarrhea in hospitalized patients receiving long-term antibiotic treatment (Rupnik et al., 2009). Moreover, *C. difficile* is the major cause of nosocomial antibiotic-associated diarrhea through the production of toxin A (TcdA) and toxin B (TcdB). The CDI disease pattern ranges from mild diarrhea to pseudomembranous colitis, toxic megacolon or colon perforation. To date, significantly expanding incidences and morbidity rates related to CDI have been reported since the 1980s and have been largely attributed to the presence of new hypervirulent strains called NAP1/BI/027, which have been implicated as the cause of numerous epidemics in the United States, Canada, Europe, and Asia (Rupnik et al., 2009), provoking greater concern regarding this bacterium worldwide (Hung et al., 2012). Although the pathogenesis of *C. difficile* has been largely attributed to the production of TcdA and TcdB by the bacteria, vaccination with these toxins has not provided complete protection against the disease in animals (Torres et al., 1995), which suggests that other factors contribute to the disease severity. The clinical outcome of CDI is defined as the result of the formation of volcano-like inflammatory effects. However, the type of protective immunity required for the prevention of *C. difficile* infection remains unclear. We hypothesized that CDI may cause innate inflammasome activation within the gastrointestinal tract. In our previous work, patients with the TLR4 rs1927914 polymorphism (GG genotype) had a higher risk of *C. difficile* colonization (Hung et al., 2013), suggesting that CDI is associated with the innate immune status of the host. Notably, various proinflammatory cytokines, such as IL-1β, are detected in patients with CDI (Steiner et al., 1997; Steele et al., 2012).

The host innate immune system is the first line of defense against microbial infection and is activated by the engagement of pattern-recognition receptors (PRRs) that are responsible for recognizing specific components expressed by the microbes (Monie et al., 2009). However, although the intracellular mechanism of the *C. difficile* toxin is well-understood, the interactions of this pathogen, either directly or indirectly, with the host innate and adaptive immune system are poorly understood. The Toll-like receptor (TLR) family represents some of the most common PRRs, which can induce downstream MyD88-mediated signaling pathways and finally activate NF-κB to induce the production of inflammatory cytokines such as TNF-α and IL-1β, which are important for pathogen clearance (Takeda and Akira, 2004; Kaisho and Akira, 2006). By contrast, Nod-like receptors (NLRs) serve as sensors of intracellular microbial invasion or stress signals and usually assemble into a large multiprotein complex called the inflammasome (Franchi et al., 2009; Martinon et al., 2009; Schroder and Tschopp, 2010). The inflammasome is a caspase-1 activation platform that regulates immune responses and disease pathogenesis (Franchi et al., 2009). Following inflammasome activation, activated caspase-1, which is a cysteine protease, can proteolytically cleave cytokines from their precursors, such as the pro-form of IL-1β (pro-IL-1β), into bioactive forms (Schroder and Tschopp, 2010; Henao-Mejia et al., 2012).

Ng et al. were the first researchers to demonstrate that inflammasome activation is also involved in *C. difficile* infection (Ng et al., 2010). Their results demonstrated that the *C. difficile* toxin can trigger IL-1β release by activating an ASC-containing inflammasome. Moreover, *ASC*^−/−^ mice and pretreatment with the IL-1 receptor antagonist Anakira ameliorated disease progression, suggesting that toxin-induced inflammation and intestinal injury are mediated by the inflammasome. In addition, *C. difficile* stimulates intracellular Nod1 signaling which triggers a positive feedback loop of neutrophil recruitment and IL-1β production (Hasegawa et al., 2011, 2012). However, the precise mechanism and regulation between intracellular *C. difficile* and the inflammasome are still unclear and controversial.

Recently, emerging studies have demonstrated that caspase-1 might be involved in another effector mechanism, referred to as pyroptotic cell death, apart from the regulation of inflammasome activation (Lamkanfi, 2011). Pyroptosis is a caspase-1-dependent specialized form of cell death that is usually induced in immune cells during infection, especially in macrophages and dendritic cells. Although certain mechanistic aspects of pyroptosis have been investigated *in vitro* during various bacterial infections (Sutterwala et al., 2007; Cervantes et al., 2008; Silveira and Zamboni, 2010), its function *in vivo* remains unclear. Remarkably, Miao et al. demonstrated that caspase-1-dependent pyroptosis promoted the clearance of intracellular bacteria *in vivo* independently of IL-1β and IL-18, suggesting that pyroptosis might serve an efficient role in host defense mechanisms (Miao et al., 2010). To date, the role of pyroptosis in *C. difficile* infection is poorly understood. Previously, Ng et al. showed that lactate dehydrogenase (LDH) release, as an indicator of pyroptosis, was elevated in LPS-primed peritoneal macrophages treated with toxins (Ng et al., 2010). However, the detailed mechanism and physiological function of *C. difficile* infection *in vivo* requires further investigation.

In conclusion, the purpose of this study was to assess the role of the inflammasome and pyroptosis during *C. difficile* infection. The mechanistic studies were conducted using macrophages infected with *C. difficile*. Additionally, caspase-1-specific inhibition was performed *in vivo* to examine the role of the inflammasome in host defense during CDI.

## Materials and methods

### Bacterial strains

Three reference strains of *C. difficile* were used in this study: the toxigenic strain VPI 10463 (*tcdA*^+^, *tcdB*^+^), the non-toxigenic strain CCUG 37780 (*tcdA*^−^, *tcdB*^−^) and BAA 1805 (*tcdA*^+^, *tcdB*^+^, *tcdC* deletion, *ctdA*^+^, *ctdB*^+^). BAA 1805 is also referred to as NAP1/BI/027, characterized as secreting large amounts of TcdA and TcdB, and referred to as the epidemic, hypervirulent strain (McDonald et al., 2005; O'Connor et al., 2009). These strains of *C. difficile* were grown anaerobically in brain heart infusion broth (Becton Dickinson, Cockeysville, MD) supplemented with 5 mg/ml yeast extract (MO BIO) and 0.1% L-cysteine (AMRESCO®) at 37°C for 2 days. The bacteria were washed twice with 1 × PBS prior to macrophage infection. To obtain heat-killed *C. difficile*, washed bacteria were suspended in 1 × PBS and incubated at 121°C for 30 min.

### Mice

Wild-type (WT) C57BL/6 mice were obtained from NCKU Laboratory Animal Center. *MyD88*^−/−^, *Tlr2*^−/−^, *Tlr4*^−/−^, and *Tlr2*^−/−^*/Tlr4*^−/−^ mice on the C57BL/6 background were maintained under conditions compliant with the rules and guideline of the National Laboratory Animal Center in Taiwan. *NLRP3*^−/−^ mice on the C57BL/6 background were kindly provided by the laboratory of Cheng-Yeu Wu and obtained from the Chang Gung University Laboratory Animal Center. All animal studies were performed according to protocols approved by the Institutional Animal Care and Use Committee of National Cheng Kung University (NCKU IACUC approval number 102296 and 100143).

### Cell culture

Peritoneal macrophages were harvested from C57BL/6 WT mice after intraperitoneal injection of 3% thioglycollate (BD Biosciences, Franklin Lakes, NJ, USA) for 2 days. The collected peritoneal lavage fluid was centrifuged, and the erythrocytes in the cell pellets were lysed using RBC lysis buffer (pH 7.3). The remaining cells were resuspended in RPMI 1640 medium (containing 10% FBS, 0.1 mg/ml kanamycin and streptomycin) and seeded at 4 × 10^6^ cells/ml in a 6-well plate. After 3–4 h, the unattached cells were washed away with 1 × PBS, and the fresh culture medium was incubated overnight for further infection experiments.

### *In vitro* cell infection

Thioglycollate-elicited peritoneal macrophages were prepared as described above. Bacteria were harvested as previously described and adjusted to the desired cell numbers in serum-free RPMI1640 medium. The cells were washed twice with 1 × PBS and infected at a multiplicity of infection (MOI) of 2 for 6 h under aerobic condition (37°C, 5% CO_2_). For the inhibition studies, the cells were co-treated for 6 h with Ac-YVAD-cmk (100, 200 μM; Cayman Chemical, Ann Arbor, MI, USA), glyburide (100 μM; Sigma-Aldrich, St Louis, MO, USA), cytochalasin D (1 μM; Sigma-Aldrich, St Louis, MO, USA), A438079 (200 μM; Tocris Bioscience, Ellisville, MO, USA), or Apyrase (5 units/ml; Sigma-Aldrich, St Louis, MO, USA). In addition, A438079 pre-treatment was applied for an additional 30 min. After 6 h of infection, the cell culture supernatants and cell protein lysates were collected and analyzed by Western blotting and/or ELISA to detect caspase-1 and IL-1β.

### siRNA transfection

siRNA of P2X7 (sc-42575) and Pryin (sc-106466) were purchased from Santa Cruz (Dallas, TX, USA), and control scramble siRNA (5860029551) was purchased from MDBio (Taipei, Taiwan). NLRP3 knockdown in THP-1 was done using lentiviral transduction. Human shNLRP3 (TRCN0000062727) clone and control LKO.1 (TRCN0000062726) were obtained from the National RNAi Core Facility (Institute of Molecular Biology/Genomic Research Center, Academia Sinica, Taiwan). THP-1 cells were transfected with siRNAs at concentration of 100 nM using the Lipofectamine 3000 (Invitrogen, Carlsbad, CA, USA) for 48 h. Then THP-1 cells were differentiated with 100 nM PMA (Sigma-Aldrich, St. Louis, Missouri, USA) for 24 h for further use.

### Assessment of extracellular ATP concentration

The concentration of ATP was measured in supernatants by using CellTiter-Glo® Luminescence Assay (Promega, Madison, WI, USA) according to manufacturer's instructions, and light production was measured by Modulus™ II microplate multimode reader (Promega, Madison, WI, USA).

### Immunoblotting

Total proteins were separated by SDS-PAGE, transferred to PVDF membranes, and probed with antibodies against IL-1β (#AF-401-NA, R&D system, Minneapolis, MN, USA), precursor and p10 subunit of caspase-1 (#sc-514, Santa Cruz, Dallas, TX, USA), NLRP3 (#AG-20B-0014, AdipoGen, San Diego, CA, USA), HMGB-1 (#AB79823, Abcam, Cambridge, MA, USA), P2X_7_ (#sc-514962, Santa Cruz, Dallas, TX, USA), pyrin (#sc-390938, Santa Cruz, Dallas, TX, USA) and α-tubulin (#T5168, Sigma-Aldrich, St. Louis, Missouri, USA). The expression of low-molecular-weight (LMW) surface layer proteins in the cell culture supernatant was also detected using rabbit anti-LMW SLP BAA 1805 serum (customized by Abnova, Taipei, Taiwan).

### Assessment of cytokine production and serum amyloid A

The levels of IL-1β, IL-18, and TNF-α in the cell culture supernatants or colon homogenates were detected using ELISA kits (eBioscience, San Diego, CA, USA) according to the manufacturer's instructions. Serum amyloid A (SAA), which has been described as an indicator of colitis, was measured using an ELISA kit (Invitrogen, Carlsbad, CA, USA) according to the manufacturer's instructions.

### Cytotoxicity assay

Lactate dehydrogenase (LDH) release from damaged cells was analyzed using a CytoTox96 Non-Radioactive Cytotoxicity Assay (Promega, Madison, WI, USA) according to the manufacturer's instructions.

### RNA isolation and real-time PCR

Total RNA was extracted using REzol (PROtech, Taipei, Taiwan). mRNA levels were analyzed with real-time quantitative RT-PCR (Applied Biosystems, Foster City, CA) with *Actb* as the reference gene in each reaction. The sequences of the primers are shown in Table 1.

**Table 1:** Sequences of the real-time primers.

**Gene symbol**	**Name**		**Sequences**
*Il1b*	IL-1β	Forward	GCA ACT GTT CCT GAA CTC AAC T
		Reverse	ATC TTT TGG GGT CCG TCA AT
*Actb*	β-actin	Forward	ACT GCC GCA TCC TCT TCC TC
		Reverse	TGC CAC AGG ATT CCA TAC CC
*Cxcl1*	CXCL1	Forward	GTT GAA GGT GTT GCC CTC AG
		Reverse	TTG GGG ACA CCT TTT AGC ATC
*Cxcl2*	CXCL2	Forward	TGT CAA TGC CTG AAG ACC CTG CC
		Reverse	AAC TTT TTG ACC GCC CTT GAG AGT GG

### *C. difficile* infection in mice

WT C57BL/6 mice received an antibiotic mixture (0.045 mg/ml vancomycin, 0.215 mg/ml metronidazole, 0.4 mg/ml kanamycin, 0.035 mg/ml gentamicin, 850 U/ml colistin, and 0.03 mg/ml clindamycin) (Sigma-Aldrich) in their drinking water for 2 days. The following day, the vancomycin and metronidazole treatment was removed to avoid killing *C. difficile*. On the day of infection, all of the mice received clindamycin (4 mg/kg; Sigma-Aldrich) intraperitoneally and were then challenged via oral gavage with *C. difficile* strain VPI 10463 (3.5 × 10^6^ CFU). The vehicle group was challenged with 1 × PBS. The mice were weighed until they were sacrificed at 48 h after challenge. CDI disease progression was defined by body weight loss, colon length, and cecum weight. All of the mice received treatments similar to those described above and were injected intraperitoneally with the caspase-1-specific inhibitor, Ac-YVAD-cmk (12.5 μM diluted in 200 μl PBS) prior to challenge with *C. difficile*. For *C. difficile* CFU to be counted in the feces, fecal pallets including stool and cecum content were collected and cultured anaerobically on *C. difficile* selective plates (Cycloserine Cefoxitin Fructose Agar, CCFA) for 48 h. The whole colon tissues were collected from mice and homogenized with lysis buffer (50 mM Tris-HCl, pH 7.6, 150 mM NaCl, 1% NP40, 0.25% sodium deoxycholate and 1 mM EDTA) to extract total protein. The amount of total extracted protein was determined by Bradford assay (Bio-rad, Munchen, Germany). The amount of IL-1β in the colon homogenate were detected using ELISA kits as described above.

### Immunofluorescence staining

Colons were fixed in 4% paraformaldehyde, and embedded in paraffin, sectioned (5 μM), deparaffinized, blocked, and incubated overnight with primary antibody against Gr-1 (#ab25377, Abcam, Cambridge, MA, USA). Secondary antibody staining was performed using AlexaFlour 488 goat anti-rat IgG (Invitrogen, Carlsbad, CA, USA) for 60 min. All slides were counterstained with DAPI (Invitrogen, Carlsbad, CA, USA).

### Statistical analysis

Values are presented as the mean ± SEM. Statistical analyses were conducted by Student's *t*-test or one-way ANOVA followed by Dunnett's multiple comparison test. Differences were considered statistically significant when the *p*-value was <0.05.

## Results

### *C. difficile* induces caspase-1-dependent IL-1β production

**Figure 1:**
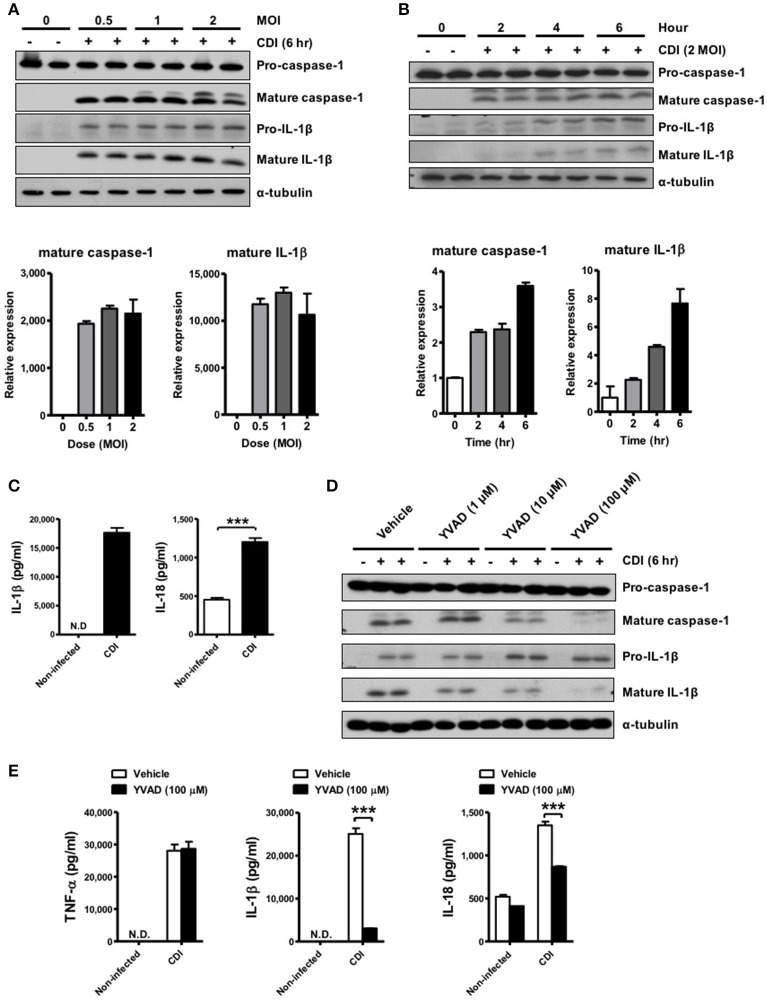
*C. difficile* induces caspase-1-dependent IL-1β production. **(A,B)** Peritoneal macrophages were infected with *C. difficile* VPI 10463 at increasing MOIs and a time course of infection was evaluated. Mature IL-1β and caspase-1 processing were analyzed by Western blotting. Lower panels showed quantification of mature caspase-1 and IL-1β relative protein level compared with non-infected group. **(C)** IL-1β and IL-18 production in the supernatant of infected cells were measured by ELISA. **(D)** Influence of the caspase-1 inhibitor Ac-YVAD-cmk (YVAD, 100 μM) on IL-1β release in infected macrophages by Western blot analysis. **(E)** The levels of secreted of IL-1β, IL-18, and TNF-α were determined by ELISA after treatment with YVAD. Values represent the mean ± SEM (*N* = 3/group). ^***^p < 0.001. N.D., not detected.

To investigate whether *C. difficile* could induce inflammasome activation, the elicited peritoneal macrophages isolated from WT mice were challenged with *C. difficile* at different MOIs, and a time course analysis was performed. The results showed that mature IL-1β and caspase-1 were obviously observed at an MOI of 0.5 for 6 h and increased in a time-dependent manner in the supernatants of the infected macrophages (Figures 1A,B). Quantified results by densitometer were shown in the lower panel. In addition, the production of IL-1β and IL-18 increased significantly after 6 h of *C. difficile* infection (Figure 1C). To demonstrate the role of caspase-1 in *C. difficile*-induced IL-1β release, we further infected the macrophages in the presence of Ac-YVAD-cmk, an irreversible specific caspase-1 inhibitor, and found that this procedure remarkably abolished the release of IL-1β and caspase-1 activation (Figure 1D). In addition, a comparable level of TNF-α production was measured by ELISA, whereas the production of IL-1β and IL-18 production were significantly reduced following caspase-1 inhibition (Figure 1E). Collectively, these data suggest that *C. difficile* induced IL-1β production is caspase-1-dependent.

### ATP-P2X_7_ pathway is essential for *C. difficile*-induced inflammasome activation

**Figure 2:**
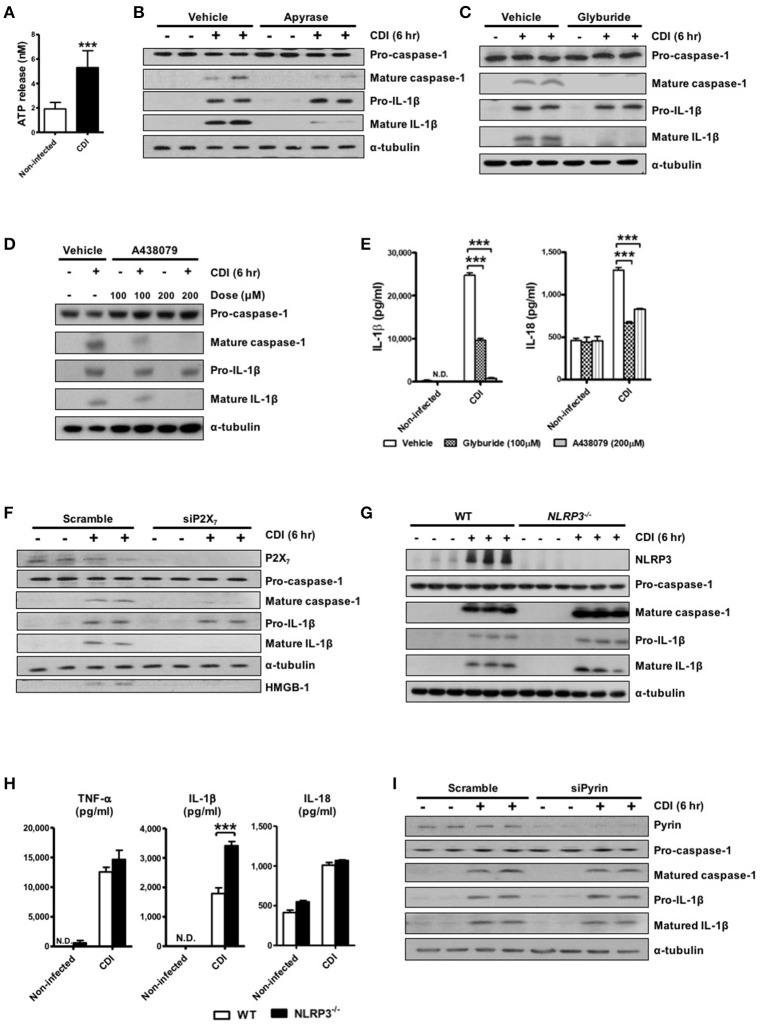
ATP-P2X_7_ pathway is essential for *C. difficile*-induced inflammasome activation. **(A)** ATP quantification in peritoneal macrophage supernatant after *C. difficile* VPI 10463 infection. **(B–D)** Peritoneal macrophages were infected with *C. difficile* VPI 10463 in the presence of apyrase (5 units/ml), glyburide (100 μM) or a 30-min pretreatment with the P2X_7_ antagonist (A438079) for 6 h. ^***^p < 0.001. Inflammasome activation, including IL-1β and caspase-1 processing, was analyzed by Western blotting. **(E)** The levels of IL-1β and IL-18 in the supernatant of infected cells after inhibitor treatment were determined by ELISA. ^***^p < 0.001, compared with vehicle-treated infected group. **(F)** Western blot analysis of inflammasome activation after transfection with scrambled siRNA or P2X_7_ siRNA in infected THP-1 cells. **(G)** IL-1β production and caspase-1 activation between WT and *NLRP3*^−/−^ infected cells were detected by Western blotting. **(H)** The secretion of IL-1β, IL-18, and TNF-α were monitored by ELISA. Values represent the mean ± SEM (*N* = 3/group). ^***^p < 0.001. N.D., not detected. **(I)** Western blot analysis of inflammasome activation after transfection with scrambled siRNA or Pyrin siRNA in infected THP-1 cells.

Robust caspase-1-dependent IL-1β production in response to *C. difficile* infection suggests the involvement of inflammasome activation. To date, various inflammasome members have been identified, among which NLRP3 and NLRC4 are the most common inflammasomes of the host innate immune system in sensing and reacting to bacterial infection (van de Veerdonk et al., 2011; Franchi et al., 2012). One of the common features of NLRP3 inflammasome activation in response to diverse stimuli is the signaling pathway that relies on the ATP-P2X_7_ pathway (Schroder and Tschopp, 2010). Thus, we evaluated the levels of released ATP after *C. difficile* infection. Elevated ATP releasing in culture supernatants was found after CDI (Figure 2A). In accordance with this, treatment with apyrase, an ATP/ADP-hydrolyzing enzyme, diminished *C. difficile*-induced IL-1β releasing (Figure 2B). These results indicated that *C. difficile*-induced inflammasome activation is elicited by ATP. Next, we blocked ATP-sensitive K^+^ channels in the presence of glyburide (Figure 2C) or pretreated with A438079 (Figure 2D), which functions as a P2X_7_ antagonist to clarify the mechanism by the ATP- P2X_7_ pathway during *C. difficile* infection. The results showed that IL-1β processing and caspase-1 activation were both significantly impaired by preventing K^+^ efflux or blocking P2X_7_. Additionally, the production of IL-1β and IL-18 measured by ELISA were markedly reduced following treatment with these inhibitors (Figure 2E). To confirm the dependency of P2X_7_ in *C. difficile*-mediated inflammasome activation, we performed knockdown experiments in THP-1 cells and monitored the effect of P2X_7_ depletion on the level of inflammasome activation. Relative to control cells treated with scrambled siRNA, the levels of mature caspase-1 and IL-1β were abrogated in the supernatant of cells treated with P2X_7_ siRNA after CDI (Figure 2F). These data indicate that the ATP-P2X_7_ pathway is essential for *C. difficile*-induced inflammasome activation. It is known that P2X_7_-induced potassium efflux majorly regulates NLRP3 inflammasome activation (He et al., 2016; Karmakar et al., 2016). To further confirm the involvement of NLRP3 inflammasome, we compared the effects observed in peritoneal macrophages isolated from WT and *NLRP3*^−/−^ mice. However, the results showed that inflammasome activation was still induced by *C. difficile* in both *NLRP3*^−/−^ peritoneal macrophages (Figure 2G) and NLRP3-knockdown THP-1 cells (data not shown). In addition, IL-1β was elevated, and there were no significant differences in IL-18 production in the supernatant of *NLRP3*^−/−^ macrophages (Figure 2H). Furthermore, we also investigated whether pyrin is involved in *C. difficile*-induced inflammasome activation. Knockdown of pyrin had no effect on the production of mature caspase-1 and IL-1β in response to CDI (Figure 2I). Taking together, the ATP-P2X_7_ pathway is required for *C. difficile*-induced inflammasome activation, but neither NLRP3 nor pyrin inflammasome-dependent.

### Intracellular bacterial induce robust inflammasome activation and pyroptosis during CDI

**Figure 3:**
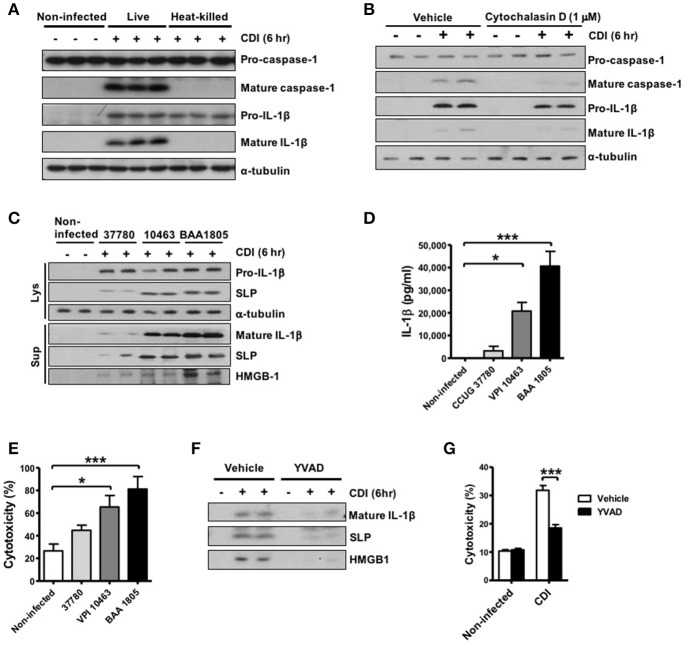
Intracellular bacterial induces robust inflammasome activation and pyroptosis during CDI. **(A)** Peritoneal macrophages were infected with live or heat-killed *C. difficile* VPI 10463 for 6 h. Mature IL-1β and caspase-1 processing were analyzed by Western blotting. **(B)** The macrophages were infected with *C. difficile* in the presence of cytochalasin D (1 μM) for 6 h. Mature IL-1β and caspase-1 processing were analyzed by Western blotting. Peritoneal macrophages were infected with different strains of *C. difficil*e (CCUG 37780, VPI 10463, and BAA 1805) for 6 h. **(C)** Pro-IL-1β and SLPs in cell lysate, and mature IL-1β, SLPs, and HMGB-1 in the supernatant of infected-macrophages were analyzed by Western blotting. **(D)** IL-1β production were assessed by ELISA. **(E)** Cell death induced by the different strains resulting in loss of membrane integrity was determined by the LDH release assay. **(F)** Mature IL-1β, SLPs, and HMGB-1 in the supernatant of infected-macrophages after caspase-1 inhibitor YVAD treatment were analyzed by Western blotting. **(G)** The LDH assay was also applied after the caspase-1 inhibitor Ac-YVAD-cmk (YVAD) treatment. Values represent the mean ± SEM (*N* = 3/group). ^*^p < 0.05; ^***^p < 0.001. N.D., not detected.

To examine the bacterial pathogen-associated molecular patterns (PAMPs) that are responsible for inflammasome activation in macrophages, we further infected cells with live and heat-killed whole bacteria of the toxigenic strain. The results showed that both live and heat-killed bacteria could induce pro-IL-1β production, but only live bacteria could induce subsequent inflammasome activation leading to IL-1β processing and caspase-1 activation (Figure 3A). The production of mature caspase-1 were almost blocked in response to the treatment with cytochalasin D (Figure 3B), which functions as an inhibitor of actin polymerization and leads to the disruption of phagocytosis, indicating that phagocytosis of the bacterium or toxin into the intracellular compartment is required to elicit inflammasome activation by *C. difficile*. To better understand the involvement of the toxin in inflammasome activation, we found that non-toxigenic strain (CCUG 37780) was sufficient to induce mature IL-1β production, but robust inflammasome activation was induced in response to toxigenic *C. difficile* infection (VPI 10463 and BAA 1805) (Figures 3C,D). Previous studies have demonstrated that inflammasome activation not only produces cytokines but also induces pyroptotic cell death, which is a specific-form of cell death induced by the infection of macrophages or dendritic cells (Bergsbaken et al., 2009; Ashida et al., 2011; Lamkanfi, 2011). However, the role of pyroptosis in *C. difficile* infection has not been clearly identified. Thus, we further investigated whether inflammasome-mediated pyroptosis is involved in CDI. We detected the loss of membrane integrity using LDH release assays and analyzed the subsequent release of intracellular contents, such as high-mobility group protein (HMGB1), as an indicator of pyroptosis. *C. difficile*-induced inflammasome activation was accompanied by elevated HMGB1 (Figure 3C) and LDH (Figure 3E) released from toxigenic strain infected macrophages. Previous studies demonstrated that pyroptosis releases intracellular bacteria to the extracellular space (Miao et al., 2010; Vande Walle and Lamkanfi, 2016). We found more surface layer proteins (SLPs) of *C. difficile* were detected in the culture supernatant with toxigenic strain infection (Figure 3C), suggesting the release of bacteria from damaged cells. Furthermore, released SLPs, HMGB1, and LDH from infected cells were completely abrogated after Ac-YVAD-cmk-induced caspase 1 inhibition (Figures 3F,G), supporting the involvement of caspase-1-mediated pyroptotic cell death in CDI.

### The critical role of MyD88 and TLR2 in *C. difficile*-induced Pro-IL-1β production

**Figure 4:**
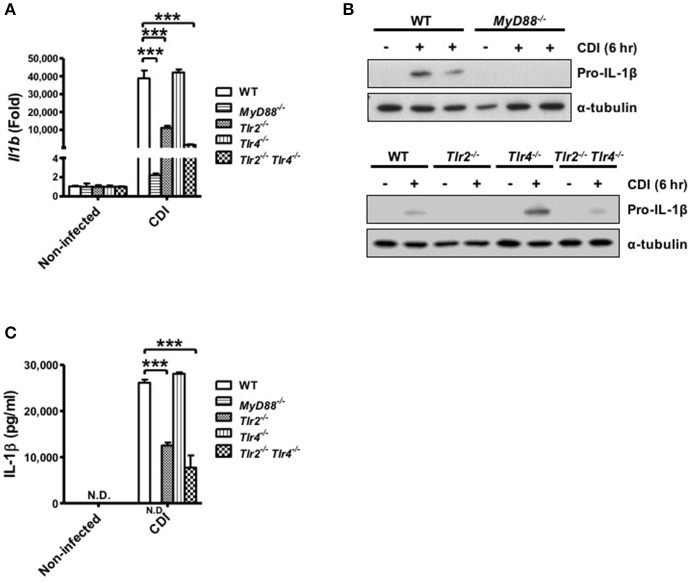
*C. difficile*-induced pro-IL-1β production is completely MyD88 and partially TLR2 dependent. Peritoneal macrophages isolated from WT, *MyD88*^−/−^, *Tlr2*^−/−^, *Tlr4*^−/−^, and *Tlr2*^−/−^*/Tlr4*^−/−^ mice were infected with *C. difficile* VPI 10463 for 6 h. **(A)** The mRNA gene expression levels of *Il1b* were determined by real-time PCR. **(B)** Pro-IL-1β production was analyzed in the cell lysate protein by Western blot analysis. **(C)** IL-1β production in the cell culture supernatant was assessed by ELISA. Values represent the mean ± SEM (*N* = 3/group). ^***^p < 0.001. N.D., not detected.

Inflammasome-mediated IL-1β secretion requires two steps: first, the engagement of TLR signaling via various proinflammatory stimuli, such as LPS, induces the pro-form of cytokines; second, activation of the inflammasome promotes mature cytokine processing (Martinon et al., 2009; Schroder and Tschopp, 2010). Thus, to analyze the role of TLR signaling in caspase-1-dependent inflammasome activation, the gene expression level of pro-IL-1β in macrophages isolated from WT, *MyD88*^−/−^, *Tlr2*^−/−^, *Tlr4*^−/−^, and *Tlr2*^−/−^*/Tlr4*^−/−^ mice was determined during *C. difficile* infection. The results showed that *Il1b* mRNA was fully reduced in *MyD88*^−/−^ and partially reduced in *Tlr2*^−/−^ and *Tlr2*^−/−^*/Tlr4*^−/−^ compared with WT macrophages, whereas the expression level in *Tlr4*^−/−^ macrophages was comparable to that in WT macrophages (Figure 4A). We further confirmed that the production pro-IL-1β protein in *MyD88*^−/−^ macrophages was completely absent and clearly reduced in infected *Tlr2*^−/−^ and *Tlr2*^−/−^*/Tlr4*^−/−^ macrophages. By contrast, there were no significant differences in pro-IL-1β production between *Tlr4*^−/−^ and WT macrophages (Figure 4B). This result is consistent with the occurrence of subsequent IL-1β secretion as assessed by ELISA (Figure 4C). In summary, these findings suggest that *C. difficile*-induced pro-IL-1β production is completely MyD88 and partially TLR2 dependent.

### *C. difficile* infection induces colonic inflammasome activation

**Figure 5:**
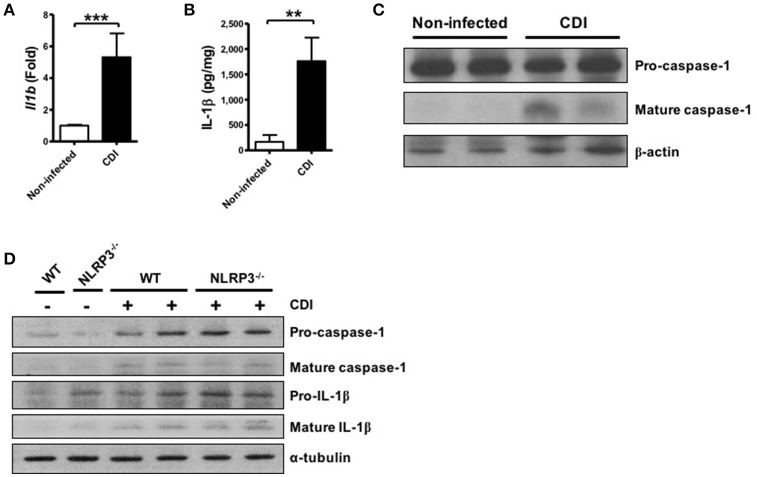
*C. difficile* infection induces colonic inflammasome activation. WT C57BL/6 mice were infected with *C. difficile* VPI 10463 for 2 days, and infected colon tissue was harvested for further analysis. **(A)** The mRNA gene expression levels of *Il1b* in the infected colon tissue were measured by real-time PCR. **(B)** IL-1β production in infected colon tissue was detected by ELISA. **(C)** Caspase-1 activation in the infected colon tissue was analyzed by Western blot. Values represent the mean ± SEM (*N* = 15/group). ^**^p < 0.01; ^***^p < 0.001. **(D)** Mature caspase-1 and IL-1β production in the colon tissue of WT and NLRP3^−/−^ mice with VPI 10463 infection were analyzed by Western blotting.

In addition to investigating the mechanism of inflammasome activation in *C. difficile* infected macrophages, we assessed the role of the inflammasome in host defense using *C. difficile*-infected mice. After 2 days of infection, infected colon tissues were harvested to analyze the level of inflammasome activation. First, we found that the gene expression level of *Il1b* was significantly increased following *C. difficile* infection (Figure 5A). In addition, not only the production of IL-1β protein but also caspase-1 activation was significantly elevated in the colon tissue of infected mice after infection (Figures 5B,C). Furthermore, we compared the effects in the colon tissue from WT and *NLRP3*^−/−^ mice. Inflammasome activation was still induced in the colon tissue of *NLRP3*^−/−^ mice during *C. difficile* infection (Figure 5D). Taken together, these findings confirmed that inflammasome activation was induced during *C. difficile* infection *in vivo*.

### Inhibition of caspase-1-dependent inflammasome activation results in more severe disease progression during CDI

**Figure 6:**
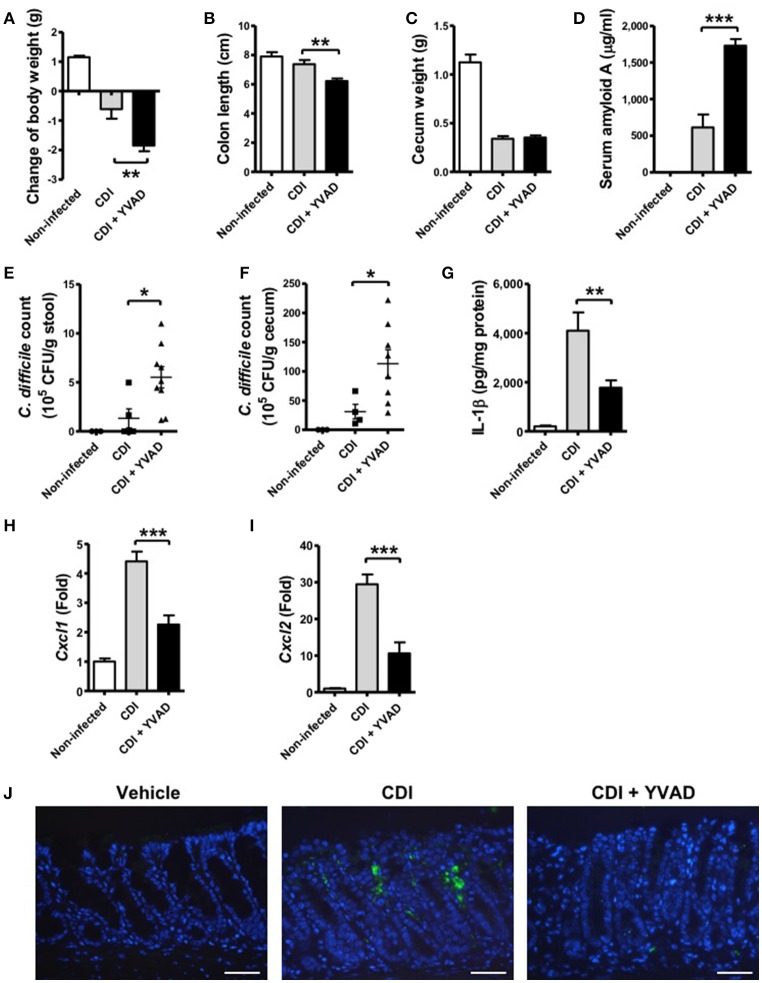
Inhibition of caspase-1-dependent inflammasome activation results in more severe disease progression during CDI. Mice were challenged with *C. difficile* VPI 10463 alone or together with an intraperitoneal injection of Ac-YVAD-cmk (12.5 μM), which is an irreversible caspase-1 inhibitor, for 2 days. Disease progression, including changes in **(A)** body weight, **(B)** colon length, and **(C)** cecum weight were monitored after 2 days of infection. **(D)** Serum amyloid A, which is an indicator of colitis, was measured by ELISA. The bacterial load of *C. difficile* in the feces **(E)** and cecum contents **(F)** were determined by plating on selective CCFA agar plates. **(G)** IL-1β production in the colon tissue was detected by ELISA. CXCL1 **(H)** and CXCL2 **(I)** mRNA levels in the colon tissue were analyzed by real-time PCR. Values represent the mean ± SEM (*N* = 5/group). ^*^p < 0.05; ^**^p < 0.01, ^***^p < 0.001. **(J)** Gr-1 positive cells in the colon tissue. Scale bar, 40 μM.

To further understand the physiological function of the caspase-1-dependent inflammasome in host defense during CDI, we examined its role via the administration of a caspase-1 inhibitor. Interestingly, the results showed that the effect of caspase-1 inhibition increased the symptom of disease, including loss of body weight, a shortened colon length, and a decreased cecum weight (Figures 6A–C). In agreement with these findings, serum amyloid A (SAA), which is an indicator of colitis, was markedly elevated following the inhibition of inflammasome activation (Figure 6D). Furthermore, the bacterial load of *C. difficile* in the feces and the cecum contents of inhibitor-treated mice were clearly increased relative to the control (Figures 6E,F). IL-1β levels in colon tissue were decreased in response to the inhibition (Figure 6G). IL-1β has been shown to be protective against infections by upregulating chemotactic chemokines and recruiting neutrophils to inflammatory sites (Biondo et al., 2014; Altmeier et al., 2016). We found that decreased IL-1β production in the colon of Ac-YVAD-cmk treated infected mice was accompanied with decreased CXCL1 (Figure 6H) and CXCL2 (Figure 6I) production and impaired Gr-1-positive neutrophil recruitment (Figure 6J), suggesting IL-1β plays some roles in bacterial clearance *in vivo*. In summary, these findings suggest that inhibition of the caspase-1-dependent inflammasome results in increased disease progression during *C. difficile* infection, which indicates that the inflammasome might play an important role in controlling the bacterial load of *C. difficile* and regulate host defense.

## Discussion

In the present study, we provided evidence that toxigenic *C. difficile* induces host inflammasome activation during infection. *C. difficile*-induced pro-IL-1β production is fully MyD88 and partially TLR2 dependent. Following the engulfment of toxigenic *C. difficile* via phagocytosis into the intracellular compartment, inflammasome activation was triggered that results in subsequent caspase-1 activation and cleavage of IL-1β processing. In addition, toxigenic *C. difficile*-induced inflammasome activation is through NLRP3-independent ATP-P2X_7_ pathway. Apart from inflammasome activation, toxigenic *C. difficile* also induced caspase-1-dependent pyroptotic cell death in infected-macrophages followed by the loss of membrane integrity and the release of intracellular contents such as LDH and especially SLPs of *C. difficile*. Furthermore, we also discovered that colonic inflammasome activation was induced *in vivo* by *C. difficile* infection and that caspase-1 inhibition led to a reduction of intestinal inflammation, increased load of *C. difficile* and disease progression, suggesting that the caspase-1-dependent inflammasome plays an important role regulating host defense during *C. difficile* infection.

Although the involvement of *C. difficile* toxins leading to pathogenesis has been well-elucidated, little is known about the potential interaction between the bacterium itself and the host immune system. Recently, emerging studies have started to examine the detailed mechanism underlying this phenomenon, suggesting that *C. difficile* can induce surface and intracellular innate and adaptive immune responses. Previously, Ryan et al. found that the SLPs of *C. difficile* could be recognized by TLR4 in bone marrow-derived dendritic cell (BMDCs) and induced the subsequent activation of the immune system to promote bacterial clearance in the host (Ryan et al., 2011). Furthermore, Jarchum et al. showed that TLR5 stimulation administered by *Salmonella*-derived flagellin protects mice against acute *C. difficile* colitis (Jarchum et al., 2011), whereas Yoshino et al. found that TcdB promotes *C. difficile* flagellin-induced chemokine production via TLR5 signaling in intestinal epithelial cells (Yoshino et al., 2013). However, the TLRs that are involved in *C. difficile*-induced inflammasome activation remain unclear. In the present study, we found that pro-IL-1β production was also affected by cytochalasin D treatment, suggesting that phagocytosis of the bacterium might be crucial for pro-IL-1β production and subsequent inflammasome activation. Moreover, the MyD88-mediated signaling pathway was involved in pro-IL-1β production and that TLR2 partially played a role in the upstream recognition of bacterium but not TLR4. This discrepancy between our findings and others might be due to variation in the different bacterial components of *C. difficile* and the cell-type specific responses.

In addition to the regulation of TLR signaling, several studies have elucidated the role of inflammasome activation during CDI. Ng et al. were the first group to show that the toxins of *C. difficile* induce ASC-containing inflammasome activation that leads to host damage (Ng et al., 2010). Xu et al further demonstrated that TcdB can trigger pyrin inflammasome activation through inactivation of Rho GTPases (Xu et al., 2014). Hasegaw et al. demonstrated that *C. difficile* could be recognized through the NOD1 signaling pathway in mesothelial cells and induce the production of chemokines such as CXCL1, which is responsible for neutrophil recruitment. It could then increase the translocation of commensals (Hasegawa et al., 2011), which further induced IL-1β production in neutrophils by regulating ASC to promote the clearance of translocated commensals during infection (Hasegawa et al., 2012). In the present study, caspase-1-dependent inflammasome activation was crucial for controlling the burden of *C. difficile*, supporting its potential protective role in host defense. However, there are some discrepancies in our findings in comparison to previous studies that might be due to the use of different experimental approaches. Ileal administration of exogenous toxin purified from *C. difficile* strain NAP1/BI/027 was used in the study of Ng et al., and thus their findings might be a result of toxin-mediated inflammation and damage. In addition, short-term of treatment *in vivo* suggested that this cytotoxic effect might directly damage the intestinal epithelium and lead to acute colitis. By contrast, Hasegawa et al. used *C. difficile* strain VPI 10463, which was used in the present study, to demonstrate that NOD1-ASC signaling pathways are important for *C. difficile*-induced inflammasome activation. Overall, these findings provide a better understanding of host immune regulation of the course of disease and host susceptibility during CDI.

According to previous studies, various NLR-containing inflammasomes have been reported to be associated with bacterial infection, such as NLRP1, NLRP3, NLRC4, and NLRP6 (Schroder and Tschopp, 2010). In the present study, we focused on the whole bacteria-induced inflammasome activation. Different from toxin-mediated pyrin inflammasome activation, we found that ATP-P2X_7_ pathway, a well-known activator of NLRP3, was essential for *C. difficile*-induced inflammasome activation. IL-1β production and caspase-1 activation were still induced in NLRP3 deficient macrophages, indicating that other NLRs in addition to the NLRP3 inflammasome might be involved in the upstream regulation during CDI. Although TcdB has been found the ability to trigger pyrin inflammasome activation (Xu et al., 2014), there is no evidence indicating that P2X_7_ affects pyrin inflammasome. In advance, our results showed that knockdown of P2X_7_ can totally abrogate inflammasome signaling in response to CDI, whereas knockdown of pyrin had no effect on inflammasome signaling, suggesting pyrin inflammasome is not involved in the whole bacteria-induced inflammasome activation. It is known that P2X_7_ is responsible in NLRP1 and NLRP3 inflammsome activation, but not NLRC4 and AIM2 (Ferrari et al., 1997; Di Virgilio, 2007; Silverman et al., 2009; Ali et al., 2011; Sharma and Kanneganti, 2016). We found that toxigenic *C. difficile*-induced inflammasome activation is through NLRP3-independent ATP-P2X_7_ pathway. Based on the above analysis, we speculated that NLRP1 might play some role in CDI. Notably, this observation is consistent with a previous study reported by Jafari et al. (2013), who showed that IL-1β production was relatively unchanged in NLRP3-deficient BMDCs but was significantly abrogated in ASC-deficient cells, suggesting that ASC may be a more crucial component of *C. difficile*-induced inflammasome activation (Jafari et al., 2013). However, the detail mechanism involving in the upstream regulation requires further investigation.

It is known that not all inflammasome functions can be abrogated either by the neutralization of IL-1β or IL-18 (Lamkanfi et al., 2010). In addition, differences in host susceptibility to IL-1β, IL-18, and caspase-1-deficient mice during infection have been reported (Henry and Monack, 2007; Lamkanfi, 2011). Taken together, these findings suggest that IL-1β, IL-18, and caspase-1 may have different physiological function in the host response during infection. Because IL-1β and IL-18 are upregulated by caspase-1, caspase-1 may mediate additional mechanisms such as pyroptosis, which supports the importance of its regulation during infection. Although the functions of the inflammasome and downstream IL-1β production in response to *C. difficile* infection have been demonstrated *in vivo* in previous studies, the role of caspase-1 activation has not been previously investigated. Our present results revealed severe disease phenotype, increased *C. difficile* burden and decreased IL-1β production after the inhibition of caspase-1 during *in vivo* infection, suggesting that caspase-1-dependent inflammasome plays an important role in the host response during CDI and impaired bacterial clearance is the major cause of severe disease progression of CDI.

However, the mechanism by which caspase-1-dependent inflammasome activation regulates host defense during CDI remains unclear. In a previous study, Miao et al. suggested that caspase-1-dependent inflammasome activation accompanied by pyroptotic cell death led to the release of bacterial components during infection. This effect might further induce immune cell recruitment into the local infected site to promote bacterial clearance (Miao et al., 2010). Interestingly, our *in vitro* results showed that SLPs, which are a specific component of *C. difficile*, were released from the damaged cells via caspase-1-mediated regulation. In fact, several reports have suggested that SLPs can also induce the production of proinflammatory cytokines including TNF-α, IL-12, IL-23, and IL-1β (Péchiné et al., 2005; Ausiello et al., 2006; Bianco et al., 2011), indicating that SLPs might have the ability to modulate the immune response during CDI (Kelly and Kyne, 2011). In our results, we found the SLP levels of non-toxigenic strain (37780) in both cell lysate and supernatant were lower than other toxigenic strains while infection with the same MOI. This indicates that the infection ability of non-toxigenic strain might lower than toxigenic strains, thereby resulting reduced intracellular inflammasome activation. Thus, a more detailed evaluation *in vivo* is needed to clarify the exact role of SLPs in the pathogenesis of *C. difficile* infection.

In addition to caspase-1-mediated pyroptosis, caspase-11 also been known to involved in inflammasome activation and the initiation of pyroptosis (de Gassart and Martinon, 2015; Yi, 2017). We found that knockdown of P2X_7_ totally abrogate inflammasome activation and the release of HMGB-1 during *C. difficile* infection, indicating that inflammasome activation and pyroptosis are controlled by P2X_7_ signaling. Thus, the involvement of caspase-11 in *C. difficile* infection might not play the major role. P2X_7_-mediated pyroptosis has been known to contribute to the release of intracellular pathogens (Coutinho-Silva et al., 2007; Di Virgilio et al., 2017). Extracellular ATP acts as a danger signal activating P2X_7_, which stimulate caspase-1 inflammasome activation and subsequently pyroptosis (Karmakar et al., 2016). P2X_7_ also acts as a downstream molecular of caspase-11 to mediate pyroptosis in response to Gram-negative pathogens (Yang et al., 2015). In addition, P2X_7_ can trigger intracellular antimicrobial responses, such as increased ROS production and phagosome-lysosome fusion to improve intracellular pathogen clearance (Moreira-Souza et al., 2017). In this study, we found the extracellular ATP engaged P2X_7_ signaling leading to NLRP3-independent inflammasome activation and pyroptosis during *C. difficile* infection.

In conclusion, we observed that caspase-1-dependent inflammasome activation was induced during toxigenic *C. difficile* infection and may act as an important regulator of host defense. Overall, the results of our study contribute to an improved understanding of host immune responses to *C. difficile* infection, which may facilitate the development of host-targeted therapeutic strategies.

## Author contributions

Y-HL, Y-CC, L-KC, and Y-HC researched data, contributed to discussion, wrote manuscript. W-CK and Y-ST contributed to discussion, reviewed and edited manuscript. P-AS, H-CL, C-YW, and Y-PH provided biomaterials, and contribute to discussion. P-JT contributed to discussion, wrote manuscript, reviewed and edited manuscript.

### Conflict of interest statement

The authors declare that the research was conducted in the absence of any commercial or financial relationships that could be construed as a potential conflict of interest.
