# Corrigendum: The ATP-P2X_7_ Signaling Axis Is an Essential Sentinel for Intracellular *Clostridium difficile* Pathogen-Induced Inflammasome Activation

**DOI:** 10.3389/fcimb.2019.00260

**Published:** 2019-07-17

**Authors:** Ya-Hui Liu, Yung-Chi Chang, Liang-Kuei Chen, Po-An Su, Wen-Chien Ko, Yau-Sheng Tsai, Yi-Hsuan Chen, Hsin-Chih Lai, Cheng-Yeu Wu, Yuan-Pin Hung, Pei-Jane Tsai

**Affiliations:** ^1^Department of Medical Laboratory Science and Biotechnology, College of Medicine, National Cheng Kung University, Tainan, Taiwan; ^2^Department of Pathology, National Cheng Kung University Hospital, Tainan, Taiwan; ^3^Division of Infectious Diseases, Chi Mei Medical Center, Tainan, Taiwan; ^4^Department of Pharmacy, Chia Nan University of Pharmacy and Science, Tainan, Taiwan; ^5^Department of Internal Medicine, National Cheng Kung University Hospital, Tainan, Taiwan; ^6^Center for Infection Control, National Cheng Kung University Hospital, Tainan, Taiwan; ^7^Department of Medicine, College of Medicine, National Cheng Kung University, Tainan, Taiwan; ^8^Institute of Clinical Medicine, College of Medicine, National Cheng Kung University, Tainan, Taiwan; ^9^Cardiovascular Research Center, College of Medicine, National Cheng Kung University, Tainan, Taiwan; ^10^Department of Medical Laboratory Science and Biotechnology, Chang Gung University, Taoyaun, Taiwan; ^11^Research Center for Industry of Human Ecology, College of Human Ecology, Chang Gung University of Science and Technology, Taoyaun, Taiwan; ^12^Graduate Institute of Health Industry and Technology, College of Human Ecology, Chang Gung University of Science and Technology, Taoyaun, Taiwan; ^13^Center for Molecular and Clinical Immunology, Chang Gung University, Taoyaun, Taiwan; ^14^Research Center of Bacterial Pathogenesis, Chang Gung University, Taoyaun, Taiwan; ^15^Department of Internal Medicine, Tainan Hospital, Ministry of Health and Welfare, Tainan, Taiwan; ^16^Center of Infectious Disease and Signaling Research, National Cheng Kung University, Tainan, Taiwan

**Keywords:** *Clostridium difficile*, inflammasome activation, pyroptosis, ATP-P2X_7_ pathway, MyD88

In the published article, there was an error in affiliations 1, 7, 8, and 9. All instances of “Medical College” used in these affiliations should be “College of Medicine.”

The authors apologize for this error and state that this does not change the scientific conclusions of the article in any way. The original article has been updated.

